# GWAS Central: a comprehensive resource for the discovery and comparison of genotype and phenotype data from genome-wide association studies

**DOI:** 10.1093/nar/gkz895

**Published:** 2019-10-15

**Authors:** Tim Beck, Tom Shorter, Anthony J Brookes

**Affiliations:** 1 Department of Genetics and Genome Biology, University of Leicester, Leicester LE1 7RH, UK; 2 Health Data Research UK, University of Leicester, Leicester LE1 7RH, UK

## Abstract

The GWAS Central resource provides a toolkit for integrative access and visualization of a uniquely extensive collection of genome-wide association study data, while ensuring safe open access to prevent research participant identification. GWAS Central is the world's most comprehensive openly accessible repository of summary-level GWAS association information, providing over 70 million *P*-values for over 3800 studies investigating over 1400 unique phenotypes. The database content comprises direct submissions received from GWAS authors and consortia, in addition to actively gathered data sets from various public sources. GWAS data are discoverable from the perspective of genetic markers, genes, genome regions or phenotypes, via graphical visualizations and detailed downloadable data reports. Tested genetic markers and relevant genomic features can be visually interrogated across up to sixteen multiple association data sets in a single view using the integrated genome browser. The semantic standardization of phenotype descriptions with Medical Subject Headings and the Human Phenotype Ontology allows the precise identification of genetic variants associated with diseases, phenotypes and traits of interest. Harmonization of the phenotype descriptions used across several GWAS-related resources has extended the phenotype search capabilities to enable cross-database study discovery using a range of ontologies. GWAS Central is updated regularly and available at https://www.gwascentral.org.

## INTRODUCTION

The genome-wide association study (GWAS) is used to better our understanding of disease aetiology by detecting associations between common genetic variants and disease traits in samples from populations. The findings from GWAS have informed translational medicine from bench to bedside, from early discoveries of novel genes linked to Crohn's disease and age-related macular degeneration (1), to knowledge informed from GWAS that has contributed to new therapies for type 2 diabetes and repositioned medications used to treat psoriasis (2). The reanalysis of GWAS data has brought new clinical insight and led to novel bioinformatic methods to interpret the data, for example, large-scale meta-analyses have suggested new drug targets for treating rheumatoid arthritis (3), and bioinformatic advances in processing and interpreting GWAS summary data has enabled the detection of novel disease variants and gene loci (4).

Controlled access to individual-level GWAS data is provided by the archival depositories dbGaP (5) and EGA (6). Several repositories, such as the NHGRI-EBI GWAS Catalog (7), GWASdb v2 (8), Open Access Database of Genome-wide Association Results (OADGAR) (9) and PheGenI (10), provide open access to limited amounts of summary-level GWAS. A common feature of these summary-level resources is that their content is restricted to marker signals that exceed predefined *P*-value thresholds. The imposition of these cut-offs can result in true disease risk variants with weak association signals (11) being omitted, and prevents the identification of consistently positive markers by directly comparing the totality of signals across and within related studies. Furthermore, the use of different ontologies to describe phenotypic observations across databases makes it problematic to identify equivalent content in the absence of interfaces to harmonize ontology concepts and interpret ontology mappings. Since the human ‘phenotype’ is an umbrella term for a range of medically and semantically distinct concepts such as diseases, medical signs and symptoms, and traits, there are a broad range of ontology options to define phenotypes. Across recently published GWAS-related databases alone, five different ontologies are used: NLM’s Medical Subject Headings (MeSH), Human Phenotype Ontology (HPO) (12), Experimental Factor Ontology (EFO) (13), Disease Ontology Lite (DOLite) (14), and the International Classification of Diseases (ICD-10) (15). Table 1 shows where these ontologies are used. While partial mappings between these ontologies are publicly available, a deeper semantic harmonization process is required to translate these mappings into a phenotype querying system that provides a means for researchers to navigate and compare equivalent database entries.

**Table 1.** Database sources of GWAS phenotype data. The ontologies used by the database, the number of studies that were imported and mapped per source, and the disease focus of the database are shown. If the source provides study URLs then the ‘PhenoMap’ interface will link to individual studies, otherwise the user is directed to the database search page.

	Ontologies	No. of mapped studies	Disease	Direct study URL	Database URL
**ALSoD**	N/A	11	Amyotrophic lateral sclerosis	Yes	http://alsod.iop.kcl.ac.uk/
**DistiLD**	ICD10	1679	All	No	http://distild.jensenlab.org/
**epiGAD**	N/A	7	Epilepsy	No	http://www.epigad.org/page/show/gwas_index
**GWAS Catalog**	EFO	3823	All	Yes	https://www.ebi.ac.uk/gwas/
**GWAS Central**	MeSH	3811	All	Yes	http://www.gwascentral.org
	HPO				
**GWAS Database**	N/A	15	All	Yes	https://gwas.biosciencedbc.jp/cgi-bin/gwasdb/gwas_top.cgi
**GWASdb v2**	HPO	1958	All	No	http://jjwanglab.org/gwasdb
	DOLite				
**PhenGenI**	MeSH	2554	All	No	https://www.ncbi.nlm.nih.gov/gap/phegeni

Given the above considerations, GWAS Central was developed to provide experimental biologists with access to a comprehensive collection of GWAS summary-level data, to enable instant interrogation and visualization of unified views of the data, and to combine such displays with information about the tested markers (16). GWAS Central restricts the display of risk alleles per SNP to ensure research study participants’ privacy or informed consent is not compromised given the possibility of identifying individuals from pooled sources of summary-level data (17). Here, we describe how we have extended GWAS Central by developing a new phenotype semantic data layer which enhances the existing visualizations, data reports and outputs; provides new interfaces for harmonized phenotype searches across GWAS-related databases using a range of ontologies; and accelerates GWAS Central data curation.

## MATERIALS AND METHODS

### Data collection and curation

GWAS Central collates summary-level association data and study metadata from many sources including GWAS databases NHGRI-EBI GWAS Catalog and OADGAR, publication supplementary materials, unprompted submissions from GWAS authors, and from direct requests for data made to researchers and consortia. The original authors of the study are cited on the website and if the study was imported from a database, the study source is also cited. Across all sources of data, various formats and differing levels of detail are received. In order to ensure maximum quality and completeness of the data, we have developed an automated data processing pipeline that validates that imported markers have valid dbSNP identifiers (18) and uses the NCBI’s Entrez Programming Utilities API to obtain from PubMed the complete record for the original study publication. The study metadata and association analysis results are integrated into a flexible and coherent data model (see Database design section).

The phenotype content for each study is evaluated and the most appropriate ontology terms are applied to standardize the description of phenotypes within and between studies. For the annotation of phenotypes, we use the MeSH controlled vocabulary and HPO. The use of these ontologies ensures that the complete spectrum of GWAS phenotypes can be precisely annotated since MeSH has deep coverage of disease categories, and HPO contains extensive phenotypic abnormalities (medical signs and symptoms). The phenotype annotations were previously manually assigned, but development of a phenotype semantic data layer means that phenotype ontology mappings (see Ontology mapping section) can be leveraged for imported data annotated to alternative ontologies, thus reducing the time spent per data import and accelerating GWAS Central data releases. To enable new cross-database phenotype ontology driven searches, we separately import study, phenotype and publication data from seven recently published GWAS-related resources. Table 1 lists the database sources and their respective ontology use. In order to incorporate databases that do not use ontologies, we assign a MeSH term to the natural language phenotype description by cross-referencing the publication identifier to the GWAS Central entry, and applying the corresponding MeSH term. If a study does not exist in GWAS Central (e.g. the study was published in a non-English language journal), then a MeSH term is manually assigned.

**Figure 1.**
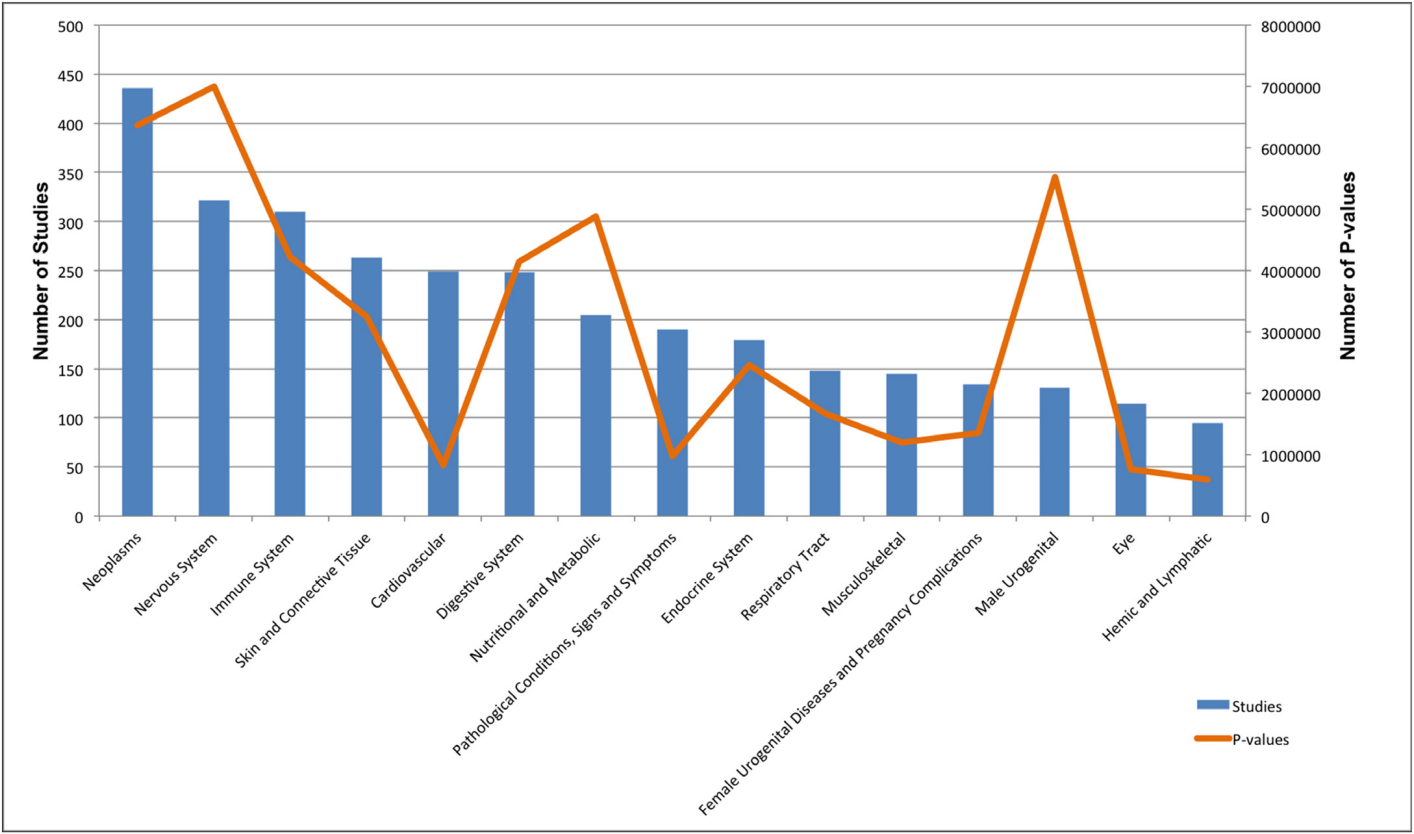
Distribution of GWAS Central data across the top 15 MeSH disease categories. *P*-value numbers refer to all submitted associations.

As of August 2019, 70566447 *P*-values from 3811 studies are available in GWAS Central, corresponding to 3251694 unique dbSNP markers and 1451 unique MeSH phenotype categories. Figure 1 shows the distribution of GWAS Central data across the most commonly studied disease areas. Cancer is the most studied disease, and the greatest number of *P*-values have been released for nervous system disorders.

### Ontology mapping

Equivalent terms within the ontologies used to annotate GWAS Central data, and to link phenotype data across databases, are formally mapped to each other. The mapped ontologies are those used by GWAS-related databases (see Table 1), namely MeSH, HPO, EFO, DOLite and ICD10. DOLite is an unstructured subset of the Disease Ontology (DO) (19), so we also use the full DO to ensure a wide coverage of mapped phenotypes. DOLite and DO share term identifiers, so phenotype content from GWASdb v2 described with DOLite was mapped to DO. Producing high-quality phenotype mappings is a challenging task and there is no publicly available comprehensive source of validated ontology mappings. Instead, there are several independent sources contributing partial ontology mappings that can disagree. We import mappings from the source ontology files that have been defined by the ontology developers (for HPO, EFO and DO), NCBO’s BioPortal (20) mappings based on close lexical matches between term names or term names and synonyms, and the EMBL-EBI Ontology Cross Reference Service mappings which include EMBL-EBI Ontology Lookup Service and Unified Medical Language System (UMLS) (21) mappings. Many of the imported mappings are not true equivalents, for example, the term *Chronic kidney disease* is mapped to the more general term *Disease*. A total of 5846 unique pairwise mappings were required to map all used terms with their equivalent terms in other ontologies. We found that all three mapping sources agreed with 1641 (28%) of the pairwise mappings, and in these cases, after manual confirmation, the two terms involved in the mapping were established to be equivalent. In future updates of this ‘PhenoMap’ content, pairwise mappings that are supported by the three sources will be assigned automatically. Mappings that were not supported by all three sources were found to be less reliable, and were manually evaluated. We will be making the final collections of ontology mappings available from the website.

### Database design

**Figure 2.**
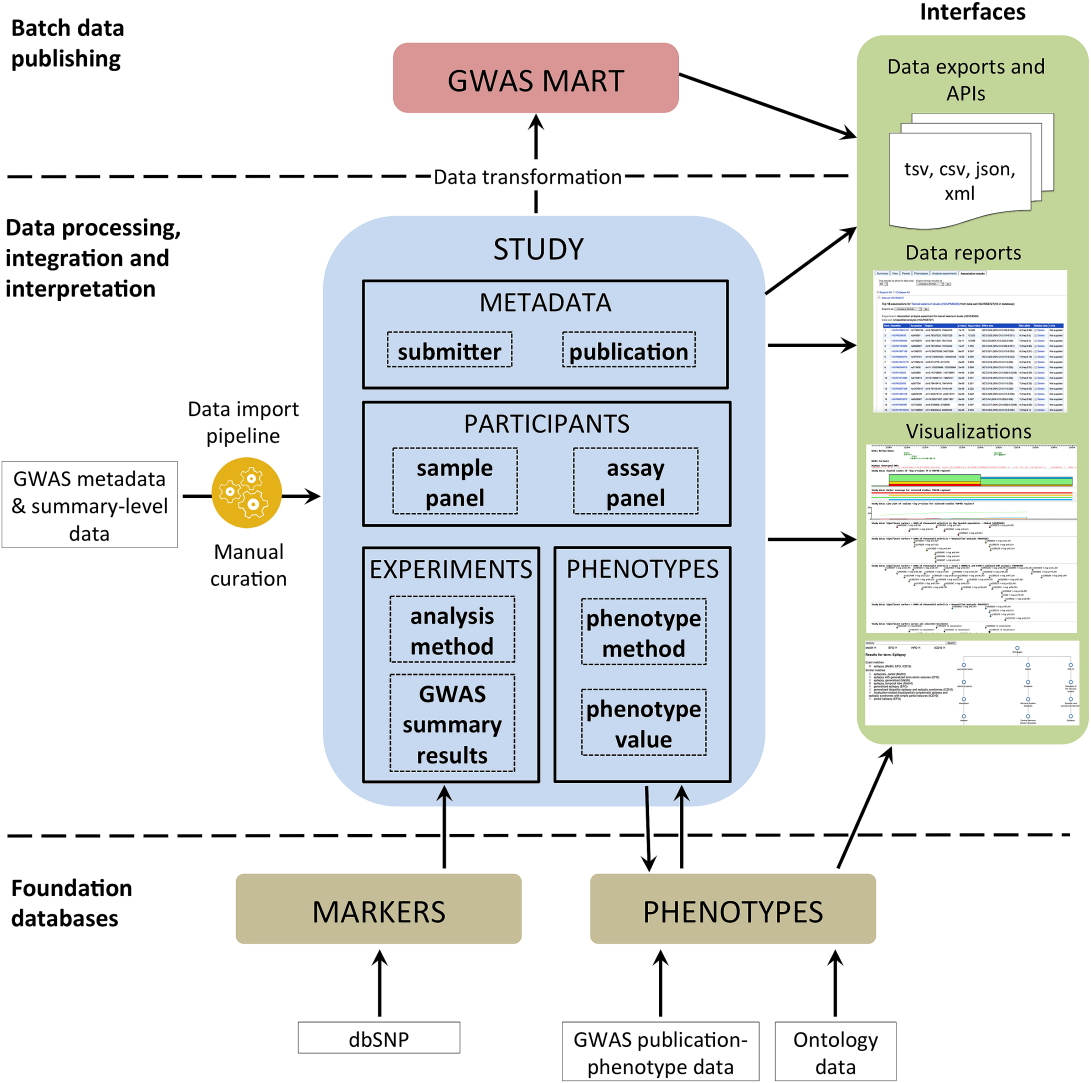
An overview of the GWAS Central system which is composed of three layers. A foundational layer of prebuilt ‘Marker’ and ‘Phenotype’ databases support the data import and curation pipelines of the ‘Study’ database, with the Phenotype database also providing ontology mappings and cross-database links via the ‘PhenoMap’ interface. The central Study database represents complex genetic association study concepts in a data model defining study metadata and participants, phenotypes, and association analysis results. Advanced search and visualization tools enable access to the Study database content. The third data aggregation layer provided by the ‘GWAS Mart’ enables advanced data interrogation across many studies. Databases are relational (MySQL) except the graph-based (neo4j) Phenotype database.

The data model underpinning GWAS Central consists of three levels as shown in Figure 2. A foundation layer of ‘Marker’ and ‘Phenotype’ databases are prebuilt. The Marker database contains core information on genetic markers from the NCBI’s dbSNP database and is used to validate data submissions to GWAS Central. The Phenotype database contains ontology data imported from source OWL, OBO and XML files, ontology mapping relationships, and links to studies and publications from GWAS Central and other GWAS-related databases (listed in Table 1). A graph database is used to provide the phenotype semantic data layer, since this enables fast real-time retrieval of complex hierarchical data structures that are otherwise difficult to model in relational systems. The central ‘Study’ database contains metadata and information about the collection of participants included in a study, each individual phenotype that is investigated by the study, and experimental details such as the analysis methods and the summary-level results generated. Data from the Study data model are transformed and loaded into the third-level data aggregation BioMart-based (22) ‘GWAS Mart’ database that provides a simplified representation of the Study database, with high data redundancy to facilitate rapid execution of complex queries.

## RESULTS

### GWAS data searching

The data discovery search box prominent on the GWAS Central front page performs queries for four types of data: (i) genes or genomic regions using HGNC gene symbols or genomic region coordinates; (ii) genetic markers using dbSNP rs identifiers or GWAS Central marker identifiers; (iii) phenotypes using MeSH or HPO terms; (iv) studies using PubMed or GWAS Central identifiers, author names or keywords in the study title and abstracts. These four data categories are also queried using the search box on the menu bar, and the search results delineate the categories. Category-specific queries, with additional tailored search options that can be adjusted by the user, are executed by selecting one of the ‘Phenotype’, ‘Gene/Region’, ‘Study list’ or ‘Marker’ tabs.

As the amount of GWAS data has increased, we have improved the phenotype-based searches to consider the progressively larger ontology directed acyclic graphs (DAGs) that must be traversed in order to retrieve study data matching a user's search criteria. The ‘is a’ relationship inheritance between ontology child and parent terms requires that all descendent terms relative to the search terms should be searched. To circumvent any delays associated with querying a relational database for complex DAG structured data, we had been preloading users’ web browsers with data in order to allow timely searches against the ontology hierarchies. This was resulting in performance delays as both GWAS Central, and the MeSH and HPO ontologies themselves, expanded following successive updates, and inevitably the MeSH and HPO DAGs used in GWAS Central phenotype data annotations increased in size. After a redesign of the phenotype search interfaces, ontology data are now loaded efficiently and dynamically from the underlying phenotype semantic data layer, enabling users to search phenotype content against query terms either typed into the query box or selected from the expandable ontology trees.

The search capabilities described here enable the discovery of matching studies, markers and associations with links to individual summaries and detailed reports. The various outputs are made available for export in a multiplicity of formats. Study summaries can be exported in JSON, XML and YAML formats, while association results can be exported in comma-, tab- or space-separated value files, RSS and Atom news feeds, RDF files, and Excel spreadsheet format. Search results and data reports are also available through REST-based web-services (technical details at https://help.gwascentral.org/web-services/). The website search tools enable results for single studies to be accessed and exported, however complex data mining and deeper data downloads in CSV and TSV file formats are available from the GWAS Mart (see Data Availability section).

### Data visualization

**Figure 3.**
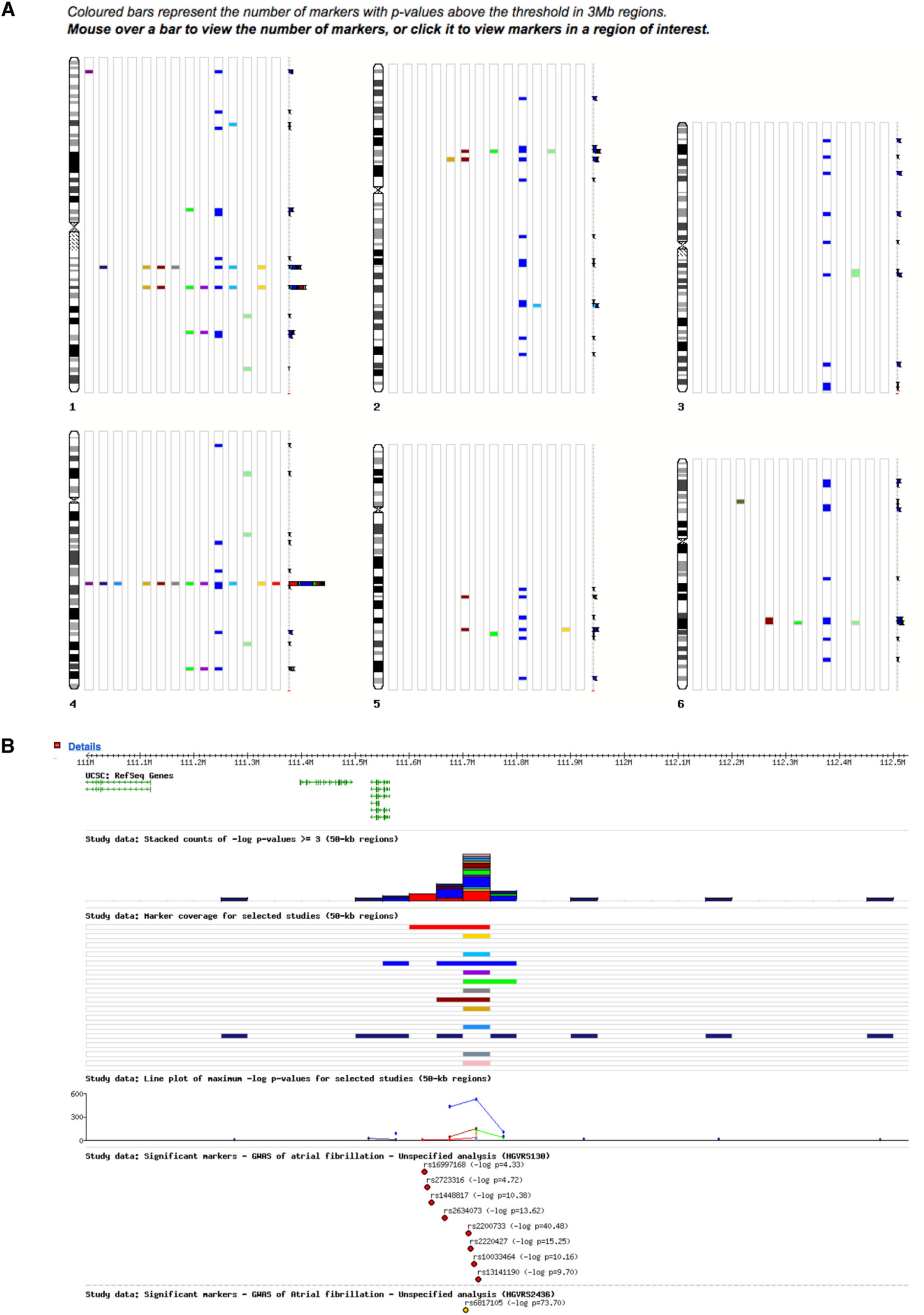
GWAS discovery and integration example using the GWAS Central genome browser. (**A**) Genome data view; marker signals from 14 atrial fibrillation data sets are plotted against all chromosomes (Chr 1–6 shown) for the selected *P*-value threshold. (**B**) Region data view; selecting a region from the genome view (in this example, the peak on Chr 4 in (A)) opens a higher resolution genome browser with optional data tracks.

A genome-wide view of each study is provided from each study report. Coloured bars plotted against each chromosome represent the relative number of markers with *P*-values exceeding the default threshold of –log ≥ 3. From this page the study can be loaded into the genome browser, from where the user can adjust the *P*-value threshold to a different level, and only associations up to that threshold are displayed. Other customizable options allow the chromosome sizes to be increased so regions with many signals can be easily delineated, and individual data sets to be plotted separately alongside the aggregated stacked plot. An objective of GWAS Central is to allow researchers to compare across multiple data sets. As such, up to sixteen study data sets can be loaded into the browser for side-by-side comparison (Figure 3). Signals of interest that have been identified in the genome-wide view can be investigated in more detail by opening that region in the higher resolution region view browser. Association markers can be viewed alongside tracks providing additional genomic features such as genes, HGMD variants and the HapMap SNP data set. A region being viewed in the browser can conveniently be opened in the UCSC and Ensembl genome browsers or exported as Excel, TSV, CSV, JSON, BED and GFF file formats.

**Figure 4.**
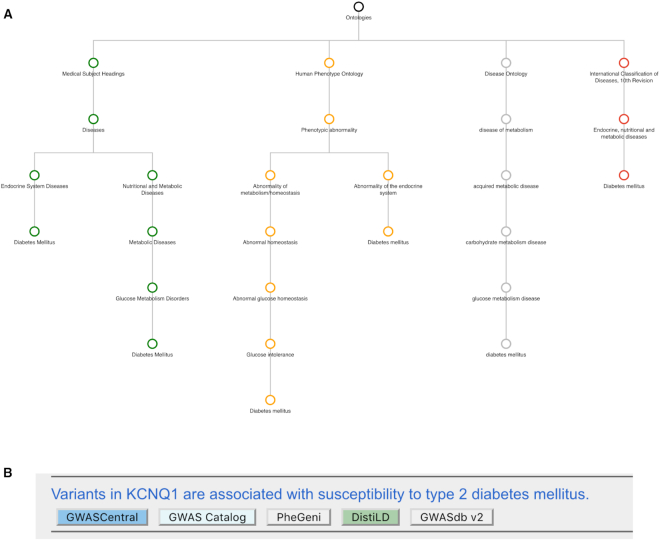
Example PhenoMap ‘term search’ outputs for *diabetes mellitus*. (**A**) The diagram panel displays all paths through the selected ontologies, from the ontology root terms to the selected terms. In this example, the MesH, HPO, DO and ICD-10 ontologies are selected, and the term nodes are colour coordinated by ontology. Selecting a node opens that node in the PhenoMap ‘browser’ for further exploration. (**B**) An example publication ‘hit’ that is annotated to the selected term. Links to the databases containing the study are provided, and selecting the publication title opens the publication in PubMed. All publications annotated to the selected terms are listed in the publication panel on both the ‘term search’ and ‘browser’ views.

The ‘PhenoMap’ tool provides unified phenotype searches across eight databases with GWAS content, enabling studies to be retrieved using every ontology used by these databases (DO includes and replaces DOLite). The tool provides two modes of interacting with ontologies: browsing an ontology hierarchy, and searching terms and synonyms. The ‘browser’ provides a hierarchy diagram of a single ontology which can be explored by expanding terms, and visualized with drag and zoom functionality. Terms are represented as nodes, and the relationships between them are displayed as edges. Selecting a node presents the GWAS publications annotated to that term, its descendent terms, and all mapped terms. Links to databases that include the identified publication/study open at the specific study page, or if the database does not support direct study linking (see Table 1), the user is directed to the database search page from where the study can be retrieved using the database's native search. A word or phrase submitted to the ‘term search’ is queried against all selected ontology terms and synonyms. As shown in Figure 4, term matches are displayed alongside hierarchy diagrams of the mapped ontologies and a list of matching publications. The diagrams and publication list are dynamically updated as terms are selected from the list of term matches. The ontology mappings can be inspected by hovering over a node of interest to highlight the mapped nodes from the other ontologies. Selecting a node from the diagram opens that node in the browser to provide deeper navigation. PhenoMap enables researchers to navigate and search a familiar ontology to retrieve studies from databases that had previously been inaccessible to that ontology.

## DISCUSSION

We work with researchers to include their studies in GWAS Central and ensure their findings are maximally represented. Many authors include a reference to their GWAS Central study entry in their manuscript, for example, Adhikari *et al* (23) cite GWAS Central study ‘HGVST3308’. GWAS Central software has been designed and optimized to handle all available summary-level data generated during a study instead of limiting the content to only a small number of ‘top’ *P*-values. Calls from Visscher *et al.* (2) and others in the field for GWAS authors to make full genome-wide summary-level findings available for their studies align with the goal of GWAS Central. For several years, GWAS Central has been periodically contacting corresponding authors of studies to request they release additional summary-level data, up to and including full genome scans. This has led to several million additional association results to be in the public domain. We publicly acknowledge any additional data received on the respective Study page, and work with authors to undertake the ‘heavy-lifting’ required to include additional data. Towards lowering the barrier to researchers making complete summary-level findings open, we provide an Excel template for authors to complete and submit to us containing their single-study metadata. We will accept summary-level data in any format researchers wish to make available to us, and our automated import pipeline will validate and integrate the findings. Future work includes making the current two-stage submission process a single step submission page on the website with real-time data validation.

Future plans to enhance the clinical utility of GWAS summary-level data include extending the semantic phenotype information to include additional ontologies, such as the systematized nomenclature of medicine clinical terms (SNOMED CT). The linking of GWAS phenotypes directly with clinical phenotypes, such as those used by the UK’s National Health Service that has adopted SNOMED CT as a coding standard, is a first and necessary step in real-time integration of clinical findings with compelling genetic variant suspects. Mouse genetic studies have the potential to validate GWAS findings (24), and recent work that we have contributed to demonstrates the capability for GWAS summary-level data to support the clinical relevance of mouse genetic screens of disease traits. For a set of mouse diabetes-related traits, we mapped the Mammalian Phenotype (MP) ontology terms describing the phenotype to the equivalent human MeSH and HPO phenotypes. The SNPs associated with the identified phenotypes in GWAS Central were compared with mouse syntenic regions, which were found to be associated with the equivalent traits in the mouse (25). Presently, human GWAS and mouse genetic study data are integrated on a limited disease or gene basis. To support genome-wide translational research involving mouse models of human disease, we will extend the semantic phenotype information to include MP and mappings between the human and mouse phenotypes. In this work, we will reuse knowledge and adopt best practices from previous research in this area, such as the cross-species ontology mappings provided by the Monarch Initiative (26), and make new mappings we produce available in the public domain.

The GWAS Central toolkit provides web-services and data outputs to enable study data to be used in remote data analyses and bioinformatics workflows, and for portions of data to be integrated and compared with external sources. For example, the DaMold data-mining platform for variant annotation (27) includes real-time GWAS Central data alongside data from other resources in its unified data aggregation interface. To promote GWAS data attribution and discovery, we contribute to the Data Citation Index on the Web of Science (28) by making available full study metadata. The metadata for each study is represented in an XML file, and we make an archive file of all metadata XMLs openly available to researchers (see Data Availability section). The study metadata files are updated after each database release. We are open to collaborating with any groups or researchers that wish to promote the wider use of GWAS Central data.

## DATA AVAILABILITY

The GWAS Central toolkit query interfaces are available from https://www.gwascentral.org. The GWAS Mart (https://mart.gwascentral.org) provides either a complete study, or 1000 markers and associated data, per download. Larger data downloads are made available to researchers who agree with GWAS Central's data sharing policy (https://help.gwascentral.org/data/data-sharing-statement/). The metadata files for all GWAS Central studies (https://help.gwascentral.org/data/gwas-central-study-meta-data/) are linked to the Data Citation Index on the Web of Science. The source code of the GWAS Central platform is available as part of a collaboration, with one such example being the nationally focused GWAS Central India (http://www.vigeyegpms.in/gwascentralindia/).
